# Serum and follicular fluid chemerin and chemerin mRNA expression in women with polycystic ovary syndrome: Systematic review and meta‐analysis

**DOI:** 10.1002/edm2.307

**Published:** 2021-10-26

**Authors:** Anahita Mansoori, Golnaz Amoochi‐Foroushani, Marzie Zilaee, Seyed Ahmad Hosseini, Maryam Azhdari

**Affiliations:** ^1^ Nutrition and Metabolic Diseases Research Center Clinical Sciences Research Institute Ahvaz Jundishapur University of Medical Sciences Ahvaz Iran; ^2^ Department of Nutrition School of Allied Medical Sciences Ahvaz Jundishapur University of Medical Sciences Ahvaz Iran; ^3^ Department of Nutrition School of Public Health Shahid Sadoughi University of Medical Sciences Yazd Iran

**Keywords:** chemerin, mRNA expression, obesity, polycystic ovary syndrome

## Abstract

**Introduction:**

Polycystic ovary syndrome (PCOS) is one of the most common endocrine disorders among women of reproductive age. Chemerin, a novel adipokine, is involved in inflammation, energy metabolism, adipogenesis, angiogenesis and insulin secretion in the adipose cells and ovary. This systematic review with meta‐analysis aimed to compare serum and follicular fluid (FF) chemerin and ovarian chemerin mRNA expression among women with PCOS and non‐PCOS.

**Methods:**

Electronic databases including Web of Science, PubMed, Google Scholar, Scopus, Cochrane and CINAHL were used for a comprehensive search through April 2021. Of the 174 articles initially identified, 22 studies met the eligibility criteria. A random‐effects model with a weighted mean difference (WMD) and 95% confidence interval (CI) was performed to compare the outcomes between groups. Subgroup and sensitivity analyses were performed to detect the sources of heterogeneity.

**Results:**

Women with PCOS compared to without PCOS showed significantly higher serum chemerin [WMD: 12.02 pg/ml (95% CI: [10.92, 13.13]), *p* < .001], chemerin mRNA expression [WMD: 0.38% (95% CI [0.25, 0.52]), *p* = .001] and FF chemerin [(WMD): 41.7 pg/ml (95% CI [17.89, 65.5]) *p* < .001]. Further, serum chemerin remained high in PCOS women even with subgroup analysis based on body mass index (BMI) or sample size (*p* < .001). Serum chemerin was higher in women with PCOS and higher BMI [(WMD): 3.29 pg/ml (95% CI: [2.73, 3.384]), *p* < .001]. The expression of chemerin mRNA was significantly higher in the PCOS group compared to the control group [WMD: 0.38% (95% CI [0.25, 0.52]), *p* < .001].

**Conclusion:**

Serum and FF chemerin and mRNA expression were higher in the PCOS group compared to the controls. Further, serum chemerin was higher in PCOS women with higher BMI compared to lower BMI. The present findings illustrate that chemerin may be associated with PCOS status and BMI, independently.

## Graphical abstract

This systematic review with meta‐analysis was shown serum, and follicular fluid chemerin levels and chemerin mRNA expression were higher in the PCOS group compared to the controls. Further, serum chemerin was higher in women with PCOS and a more BMI. The present findings illustrated that chemerin may be associated with both PCOS status and BMI, independently.

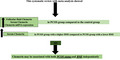

## 1 INTRODUCTION

Polycystic ovary syndrome (PCOS) is one of the most common endocrine disorders among women of reproductive age^1^ with a prevalence of 6%–10%.^2^ The clinical and metabolic disturbances of PCOS include acne, polycystic ovaries, hyperandrogenism, hirsutism, anovulation, abnormal adipokine levels, inflammation, insulin resistance (IR), glucose intolerance, obesity, type 2 diabetes mellitus, metabolic syndrome and cardiovascular risk factors, infertility and eating disorders.^3^, ^4^, ^5^

Chemerin, a novel adipokine, is encoded by gene retinoic acid receptor responder 2^6^ and synthesized as a 163‐amino acid precursor (pro‐chemerin163S)^7^ in a wide variety of cells and tissues such as placenta, lungs, skeletal muscles, kidneys, ovarian cells (granulosa cells), hepatocyte and adipocyte (brown/white).^8^ The follicular fluid (FF) chemerin levels are higher than in plasma.^9^ Chemerin with autocrine, paracrine or endocrine roles^10^ is involved in inflammation, energy metabolism, adipogenesis,^11^, ^12^ angiogenesis,^13^ failed assisted reproductive technology,^14^ and insulin secretion,^15^ IR and fat content in the adipose cells and ovary.^10^ Some studies reported that serum,^16^, ^17^ and FF^18^ chemerin levels were increased in women with PCOS.

One of the primary therapeutic strategies of PCOS is focusing on complications and metabolic disorders related to PCOS.^19^ The modification of chemerin levels in patients with PCOS may be one of the therapeutic goals of PCOS in future, due to the role of chemerin in metabolic dysfunction.^18^

A meta‐analysis recently evaluated only serum chemerin levels in women with PCOS compared to non‐PCOS (control) (both groups with normal weight). Moreover, only five studies were analysed in this regard and did not survey women with different BMI (obese or thin women).^20^ Moreover, the findings of FF chemerin levels and ovarian mRNA expression of chemerin were not pooled in the previous meta‐analyses.

Therefore, given the inconsistent and insufficient evidence (the various population (obese, normal weight, lean, with or without IR), small sample size and lack of the previous comprehensive meta‐analysis), this systematic review with meta‐analysis was aimed to evaluate serum or FF chemerin levels and also the ovarian mRNA expression of chemerin in women with PCOS in comparison with non‐PCOS.

## 2 MATERIALS AND METHODS

### 2.1 Search strategy

This literature was designed in accordance with the Preferred Reporting Items for Systematic Reviews and Meta‐Analyses (PRISMA).^21^ The registration number of our systematic review in PROSPERO is CRD42020218793. A comprehensive literature search was performed to find the relevant articles published from inception to April 2021. Two independent investigators (G.A. and M.A.) searched the electronic databases including ISI Web of Science, PubMed, Google Scholar databases, Scopus, Cochrane, Scientific Information Database (SID) and CINAHL using the combination of the following keywords:

- Polycystic ovary syndrome, polycystic ovary disease, ovary syndrome, polycystic syndrome, polycystic ovary, stein‐leventhal syndrome, stein‐leventhal, sclerocystic ovarian degeneration, ovarian degeneration, sclerocystic ovary syndrome, polycystic ovarian syndrome, ovarian syndrome, sclerocystic ovaries, sclerocystic ovary, PCOS.
- Chemerin, chemerin protein, human TIG2 protein, tazarotene induced gene‐2 protein, retinoic acid receptor responder protein 2, RAR‐responsive protein TIG2.

The search query of this review was formulated using the PICO strategy^22^ (population (P), Intervention or Exposure (I), Comparison (C), Outcome (O)) as follows: women in the reproductive age (P), PCOS (I), control (no PCOS) (C) and at least one of the variables, including serum chemerin levels, FF chemerin levels or ovarian chemerin mRNA (O).

The details of the search strategy for one of the electronic databases (PubMed) were shown in Table S1. In addition, the references cited in all selected articles were retrieved to identify further relevant publications. There was not any restriction according to the conference paper, publication year and language.

### 2.2 Inclusion and exclusion criteria

The inclusion criteria for the included studies for the present meta‐analysis were as follows: (1) observational studies (cross‐sectional, cohort and case‐control) and clinical trials (the presence of at least one of the studied variables at the baseline), (2) human studies with no restrictions on study parameters (study duration, design or sample size) and race, (3) women of reproductive age (no restriction for body mass index (BMI)) whom a diagnosis of PCOS was made according to the criteria defined by National Institutes of Health,^23^ Rotterdam,^24^ Androgen Excess Society,^25^ laparoscopic^26^ or International Classification of Diseases,^27^ (4) the studies assessed serum chemerin levels, FF chemerin levels or ovarian chemerin mRNA expression in both groups and (5) the studies in which the form of reported variables was presented in mean ± standard deviation (SD) or standard error of the mean (SEM), and median with interquartile range (IQR) or (minimum‐maximum). In the case of multiple reports for the same studied participants, the most complete data set was analysed.

Exclusion criteria included the studies which (1) compared the differences between women and men, (2) did not present reliable and sufficient data of serum chemerin, FF chemerin or ovarian chemerin mRNA expression and (3) did not have a control group.

### 2.3 Study selection

After excluding duplicate studies, two researchers (G.A. and M.A.) independently reviewed the titles and abstracts to find the relevant articles. Then, they read the full text of the included studies for selecting the eligible studies, according to the predefined criteria. Any disagreement was resolved by the discussion with a third investigator (M.Z.).

### 2.4 Data extraction

The data of the eligible studies were independently extracted by two researchers (G.A. and M.A.) in close consultation with a third researcher (M.Z.) as follows:

First author's name, year of publication, PCOS diagnostic criteria, country, type of the study, the total number of the participants, number of the participants in each group, age, serum or/and FFchemerin levels, and ovarian chemerin mRNA expression and assay approach for all the studied variables. The final data used in the meta‐analysis were rechecked to minimize the extraction errors by three researchers.

### 2.5 Quality assessment of studies

The quality of the selected studies was appraised using the Newcastle‐Ottawa scale (NOS)^28^ for all observational studies (cross‐sectional, case‐control and cohort) and the Cochrane Collaboration's tool^29^ for the randomized trials by two researchers (M.Z. and M.A.), separately. Any discrepancy was resolved by a third researcher (A.M.).

The NOS tool includes eight items in three domains with scores ranging from 0 to 9 stars. Three domains include selection, comparability and relating to the study type outcome (cohort studies) or exposure (case‐control studies). A maximum of one star in each item is allocated to the highest quality studies with the exception of one item (comparability) that is awarded two stars. The quality of the studies was categorized as low (0–3 stars), moderate (4–6 stars) and high (7–9 stars) based on total stars.^30^

The Cochrane Collaboration's tool includes six domains in seven items as follow: (1) selection bias ((two items): (a) random sequence generation and (b) allocation concealment), (2) performance bias (one item), (3) detection bias (one item), (4) attrition bias (one item), (5) reporting bias (one item) and (6) other sources of bias (one item). With respect to each item, the risk of bias was assessed as unclear (the details of the studies were not enough for the judgment) (score = 0), high (score = −1) or low (score = 1). The overall quality of each trial was categorized into low (score = −6 to 0), medium (score = 1–3) or high (score = 4–6).^29^

### 2.6 Outcome measures

The primary outcomes included the comparison of serum or FF chemerin levels and ovarian chemerin mRNA expression in women with and without PCOS.

### 2.7 Statistical analysis

We calculated the pooled estimates of weighted mean differences (WMD) and 95% confidence interval (95% CI) to evaluate the differences in the outcomes between the case and control groups. A random‐effects model was used to compare the outcomes between the groups. A chi‐square test (Cochran *Q* test) and *I* ^2^ were used to evaluate between‐study heterogeneity. Heterogeneity was considered low if *I* ^2^ < 30%, moderate if *I* ^2^ = 30–75% and high if *I* ^2^ > 75%.^31^ Subgroup analysis was performed to detect the sources of heterogeneity due to insufficient available data based on BMI (BMI < or >25 kg/m^2^) and the sample size of the studies (<30 or >30). Each study with a sample size <30 in the case or the control group was inserted in a group of sample size <30 and each study with a sample size of more than 30 (>30) in both case and control groups was inserted in a group of sample size >30. To estimate the impact of each study on the overall effect size by the leave‐one‐out method (when one study had been excluded in each turn and repeated the analysis), a sensitivity analysis was performed using influence analysis. Funnel plots were performed to indicate the presence of publication bias using Egger's regression asymmetry test. STATA 14.0 (Stata Corp.) was used for the analysis of data. Statistically, a significant difference was considered as *p* < .05.

## 3 RESULTS

### 3.1 Search result

**FIGURE 1:**
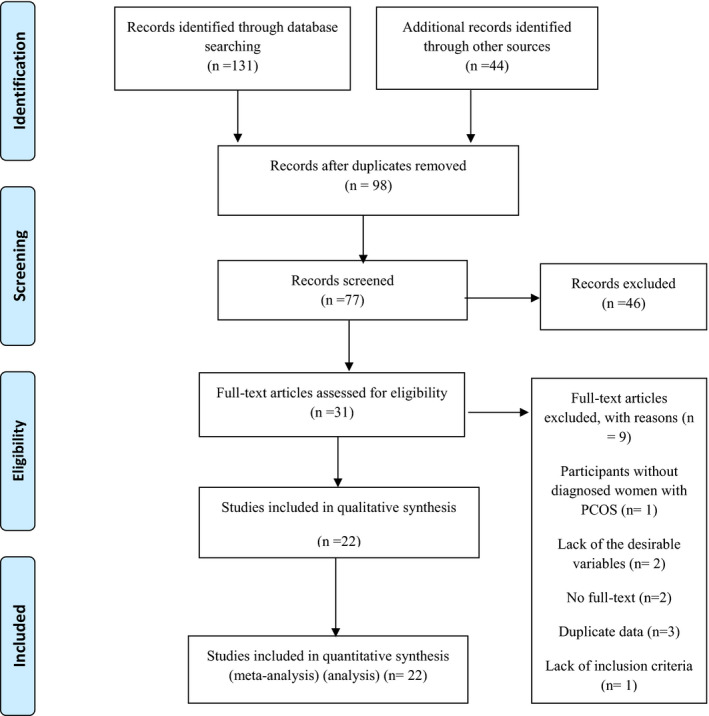
Flow diagram of the study selection process

Primary identified records through the databases and other sources searching and also flow diagram of the study selection process were presented in Figure 1 and Table S2. In total, 175 studies were preliminarily identified by the search study and the reference lists of the studies. After removing duplicates, 77 articles remained. Based on the titles and abstracts screening, 46 articles were excluded. From 31 of the selected studies for full‐text screening, 22 studies met inclusion criteria after reading the full text. GetData Graph Digitizer software was used for the data extraction of some studies.^10^, ^17^, ^18^, ^32^, ^33^, ^34^, ^35^ Some data were received by email.^35^, ^36^ The reasons for the exclusion of nine articles after reading full text were illustrated in Table S3.

### 3.2 Study characteristics

The characteristics of the 22 included studies for the present meta‐analysis were summarized in Table S4.

### 3.3 Qualitative synthesis

In total, serum and FF chemerin levels were measured in nineteen^16^, ^17^, ^18^, ^32^, ^33^, ^35^, ^36^, ^37^, ^38^, ^39^, ^40^, ^41^, ^42^, ^43^, ^44^, ^45^, ^46^, ^47^, ^48^ and four studies,^10^, ^18^, ^34^, ^49^ respectively. The methods of assessing serum and FF chemerin levels were ELISA^10^, ^16^, ^17^, ^18^, ^32^, ^33^, ^34^, ^35^, ^36^, ^37^, ^38^, ^39^, ^40^, ^41^, ^42^, ^43^, ^44^, ^45^, ^46^, ^47^, ^48^or Wb.^49^ However, in total, five studies assessed chemerin mRNA expression in the subcutaneous and omental adipose tissues^17^, ^32^ and ovarian (granulosa cells in follicular fluid)^10^, ^18^, ^34^ using reverse transcription‐polymerase chain reaction (RT‐PCR), due to the lack of data, only ovarian chemerin mRNA expression was analysed. The results of the quality assessment of the studies were shown in Tables S5and S6.

### 3.4 Quantitative synthesis

Forest plot and funnel plot diagrams and influence analysis (leave‐one‐out sensitivity analysis) on our outcomes are depicted in Figures 2, 3, 4, 5, Figures S1–S5 and Table S7. Meta‐analysis and subgroup analysis for the comparison of the outcomes between groups were projected in Table 1.

**FIGURE 2:**
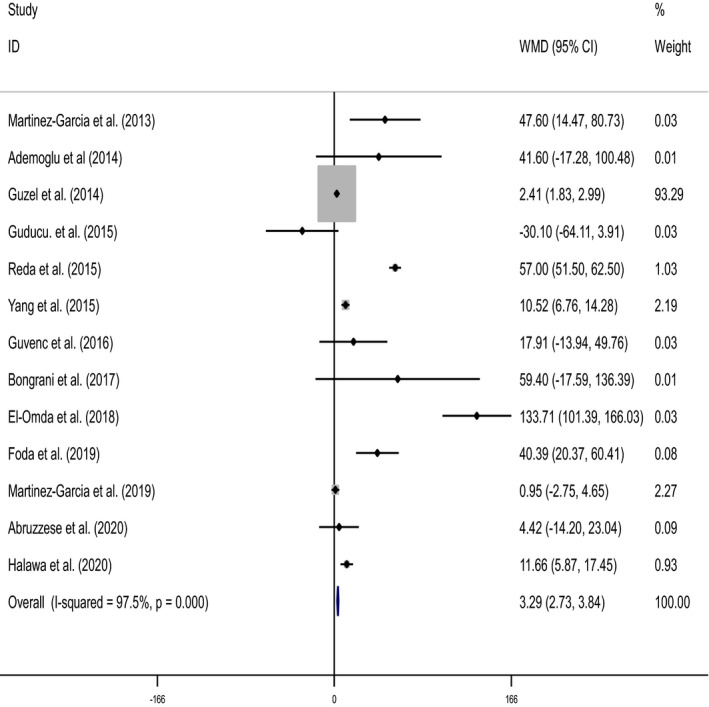
Forest plot detailing weighted mean differences (WMD) and 95% confidence intervals for the comparison of serum chemerin levels between two PCOS groups (women with the BMI >25 or 30 and PCOS women BMI <25 or 30)

**FIGURE 3:**
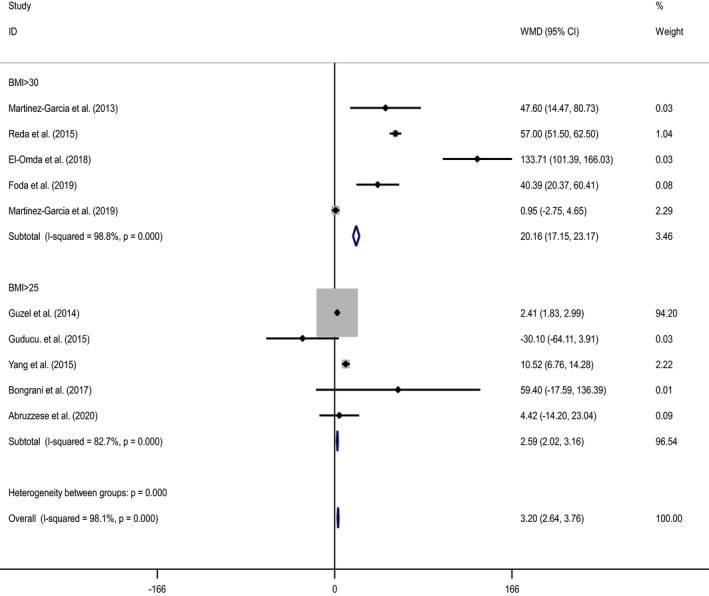
Forest plot of subgroup analysis for the comparison of serum chemerin levels in PCOS group with BMI >30 or >25 compared to PCOS group with the BMI <30 or <25

**FIGURE 4:**
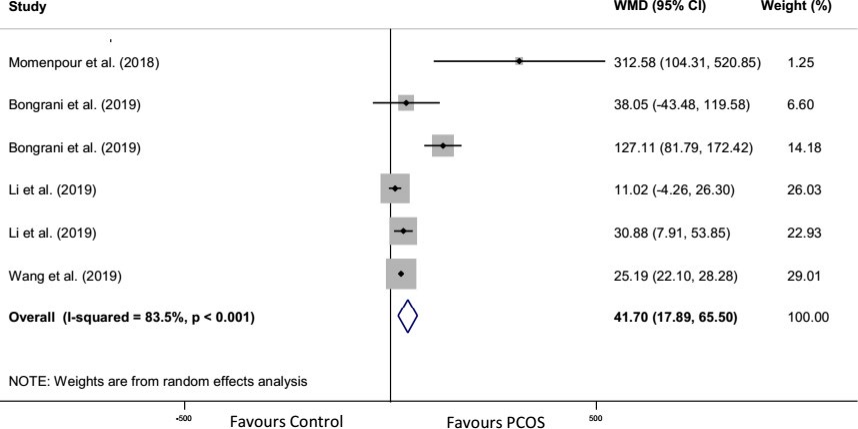
Forest plot detailing weighted mean differences (WMD) and 95% confidence intervals for the comparison of follicular fluid chemerin levels in PCOS with control group

**FIGURE 5:**
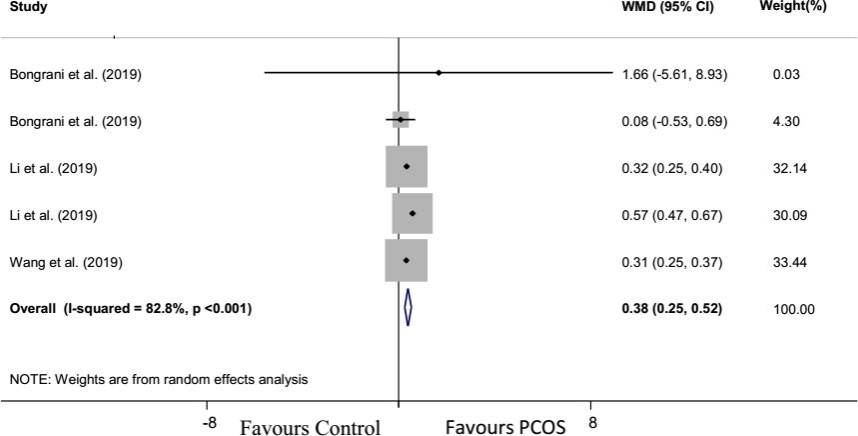
Forest plot detailing weighted mean differences (WMD) and 95% confidence intervals for the comparison of ovarian chemerin mRNA expression in PCOS with control group

**TABLE 1:** Meta‐analysis and subgroup analysis for the comparison of the studied outcomes between the studied groups

Outcomes	No. of participants (case/control)	No. of studies	Quantitative data synthesis				Heterogeneity analysis		
			WMD	95% CI	*Z*‐value	*p*‐value	df (*Q*)	*I* ^2^%	*p* value
Serum chemerin levels in PCOS and no PCOS group									
Overall effect	2256 (1191/1065)	19	12.02	10.92, 13.13	21.26	<.001	31	99.4	<.001
Subgroup analysis based on BMI (BMI >25 or <25)									
BMI >25	887 (512/375)	14	3.98	3.02, 4.9	8.23	<.001	14	98.5	<.001
BMI <25	834 (406/428)	11	0.9	0.43, 1.38	3.74	.065	11	98.1	<.001
Overall effect	1721 (918/803)	17 (	1.62	1.25, 1.99	8.51	<.001	26	98.3	<.001
Subgroup analysis based on sample size of the studies (sample size >25 or <25)^a^									
Sample size <30	774 (443, 331)	11	6.13	5.25, 7.01	13.63	<.001	16	99.5	<.001
Sample size >30	1143 (605, 538)	10	14.23	11.03, 17.43	8.71	<.001	11	99.4	<.001
Overall effect	1917 (1048, 869)	19	4.12	3.59, 4.66	15.08	<.001	28	99.3	<.001
Serum chemerin levels in PCOS group with BMI >25 or 30 and PCOS group with BMI <25 or 30									
Overall effect	800 (450/350)^c^	13	3.29	2.73, 3.84	11.55	<.001	12	97.5	<.001
Subgroup analysis based on BMI (BMI >25 or 30)									
BMI >30	247 (129/118)^d^	5	20.16	17.15, 23.17	13.12	<.001	4	98.8	<.001
BMI >25	448 (269/ 179)^e^	5	2.59	2.02, 3.16	8.91	<.001	4	82.7	<.001
Overall effect	800 (450/350)	10	3.2	2.64, 3.76	11.55	<.001	12	98.1	<.001
Follicular fluid chemerin levels in PCOS and no PCOS groups									
Overall effect	224 (114/110)	4	41.7	17.89, 65.50	3.43	.001	5	83.5	.001
Chemerin mRNA expression in PCOS and no PCOS groups									
Overall effect	204 (104/100)	3	0.38	0.25, 0.52	5.56	<.001	4	82.8	<.001

Abbreviations: 95% CI, 95% confidence intervals; BMI, body mass index; *I* ^2^, *I*‐squared; PCOS, polycystic ovary syndrome; WMD, weighted mean differences.

bSample size <30: Each study with sample size less than 30 in case or control group; Sample size >30: Each study with sample size more than 30 in both case and control group.

^a^ Considering sample size of the studies >25 or <25, serum chemerin levels compared between PCOS and no PCOS group as case and control group, respectively.

^c^ Serum chemerin levels compared between PCOS group with BMI >25 or 30 and PCOS group with BMI <25 or 30, as case and control group.

^d^ Serum chemerin levels compared between PCOS group with BMI >30 and PCOS group with BMI <30 as case and control group, respectively.

^e^ Serum chemerin levels compared between PCOS group with BMI >25 and PCOS group with BMI <25 as case and control group, respectively.

### 3.5 Meta‐analysis for serum chemerin levels

#### 3.5.1 The comparison between the PCOS and non‐PCOS groups

At first, the meta‐analysis was performed for all studies. Significant higher serum chemerin levels were seen in the PCOS group in comparison with the non‐PCOS group (WMD: 12.02 pg/ml, 95% CI: 10.92, 13.13, *p* < .001). Heterogeneity in the analysis using a random‐effect model was detected (*n* = 32, *I* ^2^ = 99.4%, *p* < .001) (Figure S1). Sensitivity analysis was performed using influence analysis through the leave‐one‐out method for all studies (Table S7); heterogeneity did not change by sensitivity analysis.

Then, subgroup analysis was carried out for all studies with BMI >25 or <25 kg/m^2^. Serum chemerin levels were higher in the PCOS group compared to the non‐PCOS group (WMD: 1.62 pg/ml, 95% CI: 1.25, 1.99, *p* < .001). Heterogeneity in the analysis using a random‐effect model was detected (*n* = 17, *I* ^2^ = 98.3%, *p* < .001; Figure S2).

In a subgroup analysis according to BMI >25 or <25 kg/m^2^, women with PCOS and BMI >25 kg/m^2^ revealed significantly higher serum chemerin levels in comparison with the non‐PCOS group with BMI >25 kg/m^2^; (WMD: 3.96 pg/ml, 95% CI: 3.02, 4.9, *p* < .001). Whereas, there was no significant difference between women with PCOS and BMI <25 kg/m^2^ compared to the non‐PCOS group with BMI <25 kg/m^2^; (WMD: 0.9 pg/ml, 95% CI: 0.43, 1.38, *p* = .065). In a subgroup analysis of serum chemerin levels, heterogeneity remained significant (*n* = 15, *I* ^2^ = 98.5%, *p* < .001) and (*n* = 12, *I* ^2^ = 98.1%, *p* < .001) in the groups with BMI >25 kg/m^2^ and BMI <25 kg/m^2^, respectively (Figure S2).

In a subgroup analysis according to sample size of the studies >30 or <30, serum chemerin levels were remarkably higher in the PCOS group in comparison with the non‐PCOS group in both studies with sample size >30 (WMD: 14.23 pg/ml, 95% CI: 11.03, 17.43, *p* < .001) and sample size <30 (WMD: 6.13 pg/ml, 95% CI: 5.25, 7.01, *p* < .001). Heterogeneity was found significant in both groups with sample sizes >30 (*n* = 16, *I* ^2^ = 99.5%, *p* < .001) and <30 (*n* = 11, *I* ^2^ = 99.4%, *p* < .001), respectively (Figure S3).

#### 3.5.2 Women with PCOS and BMI >30 or 25 kg/m^2^ and BMI <30 or 25 kg/m^2^

Women with PCOS and BMI >30 or >25 showed significantly higher levels of serum chemerin compared to women with PCOS and BMI <30 or <25 kg/m^2^; (WMD: 3.29 pg/ml, 95% CI: 2.73, 3.84, *p* < .001). Heterogeneity in the analysis using a random‐effect model was detected (*n* = 13, *I* ^2^ = 97.5%, *p* < .001) (Table 1 and Figure 2).

In a subgroup analysis based on the BMI, serum chemerin levels were higher in women with PCOS and BMI >30 kg/m^2^ than women with PCOS and BMI <30 kg/m^2^ (WMD: 20.16 pg/ml, 95% CI: 17.15, 23.17, *p* < .001). The significant heterogeneity was revealed (*n* = 5, *I* ^2^ = 98.8%, *p* < .001) (Figure 3).

Similar results were obtained when there were higher levels of serum chemerin in women with PCOS and BMI >25 kg/m^2^ compared to women with PCOS and BMI <25 kg/m^2^; (WMD: 2.59 pg/ml, 95% CI: 2.02, 3.16, *p* < .001). Heterogeneity in the analysis using a random‐effect model was detected (*n* = 5, *I* ^2^ = 82.7%, *p* < .001) (Table 1 and Figure 3).

### 3.6 Meta‐analysis for FF chemerin levels

The meta‐analysis was performed for all studies.^10^, ^18^, ^34^, ^49^ Significantly higher of FF chemerin levels were observed in the PCOS group compared to the control group (WMD: 41.7 pg/ml, 95% CI: 17.89, 65.5, *p* = .001) with the significant heterogeneity (*n* = 6, *I* ^2^ = 83.5%, *p* < .001; Figure 4).

### 3.7 Meta‐analysis for ovarian chemerin mRNA expression

The random‐effect model of meta‐analysis revealed a significantly higher expression of chemerin mRNA in the PCOS group compared to the control group (WMD: 0.38%, 95% CI: 0.25, 0.52, *p* < .001). Heterogeneity was significant in chemerin mRNA expression analysis (*n* = 5, *I* ^2^ = 82.8%; *p* < .001; Figure 5).

### 3.8 Publication bias

The funnel plot for serum chemerin levels in all studies and in the studies regarding BMI >25 or <25 was depicted in Figures S1 and S2, respectively. A significant publication bias was revealed for serum chemerin levels among studies by Egger's test in all studies and in the studies regarding BMI >25 or <25 kg/m^2^ (*p* < .001 and *p* = .018, respectively) (Table S8). The funnel plot for FF chemerin levels and ovarian chemerin mRNA expression was not performed to assess the publication bias due to the unreliable results when less than ten studies were included in a meta‐analysis.^29^

## 4 DISCUSSION

In this systematic review with a meta‐analysis of twenty‐two studies (twenty‐one observational studies and one clinical trial), the comparison of serum and FF chemerin levels, and ovarian chemerin mRNA expression was performed between the PCOS and the non‐PCOS groups. Furthermore, serum chemerin levels were compared between two PCOS groups with the different BMI categories. The findings illustrated that the levels of the studied outcomes were significantly higher in the PCOS group in comparison with the non‐PCOS group. However, serum chemerin levels were higher in women with PCOS and BMI >25 kg/m^2^compared to the women with non‐PCOS and BMI >25 kg/m^2^. Women with PCOS and BMI <25 kg/m^2^did not show any significant enhancement in serum chemerin levels compared to women with non‐ PCOS and BMI <25 kg/m^2^. Moreover, serum chemerin levels were remarkably higher in women with PCOS and higher BMI compared to women with PCOS and lower BMI. Therefore, serum chemerin levels showed an independent relationship with BMI. In agreement with the present findings, Guzel et al.^39^ showed higher serum chemerin levels in women with PCOS or women with PCOS and obesity compared to women with non‐PCOS or women with PCOS and non‐obesity, respectively. In addition, they found higher chemerin levels in women with PCOS and obesity compared to women with PCOS and normal weight. Their results showed serum chemerin levels related to fat mass more than PCOS status.^39^ The increased levels of both serum and FF chemerin were observed in women with PCOS and IR compared to the non‐PCOS with IR. Moreover, serum and FF chemerin levels were higher in PCOS with IR compared to PCOS without IR. Lie et al.^18^ illustrated that enhanced serum chemerin levels may lead to IR, the decreased GLUT4 expression and glucose uptake in the cultured hGLs. On the other hand, they pointed that the increased chemerin expression in human granulosa‐lutein cells (hGLs) may emanate from insulin.^18^

In a study conducted by Halwa et al., ^47^ they found that serum chemerin levels were the highest in women with PCOS and obesity. However, based on the previous findings,^10^, ^17^, ^18^, ^33^, ^39^, ^47^, ^50^, ^51^ some factors or metabolic status such as type 2 diabetes mellitus, impaired glucose tolerance, metabolic syndrome, PCOS, BMI, fat mass or adipose tissue, fasting insulin, and IR, serum triglycerides, and blood pressure may influence serum and FF chemerin levels or ovarian chemerin mRNA expression, the data were weak or low for other variables (subcutaneous and omental adipose tissue chemerin mRNA expression or protein expression). It was notable that all studies showed serum or FF chemerin levels are higher in women with PCOS compared to the controls with regardless of another metabolic or androgenic status (BMI, insulin, IR and lipid profiles).^14^, ^18^, ^33^, ^35^, ^39^, ^47^ The difference in the metabolic status may play a greater role in increased chemerin levels.

As the previous studies^14^, ^18^ and the present study emphasized, both serum and FF chemerin were higher in PCOS in compared to non‐PCOS. In the present study, the difference between the metabolic status of women may have led to non‐significant chemerin levels between women with PCOS and BMI <25 kg/m^2^compared to women with non‐PCOS and BMI <25 kg/m^2^.

High heterogeneity was observed in all analyses which may be related to the different races, ages, different population backgrounds, severity of the disease, and diet of the studied population and type of kit was used to evaluate the outcomes. No single study contributed to between‐study heterogeneity in subgroup and sensitivity analysis.

The function of chemerin on glucose and lipid metabolism (production of energy substrates or sources), and the pathogenesis of obesity and PCOS are controversial.^51^, ^52^ Some mechanisms can explain the cause of higher serum chemerin levels in women with PCOS as well as in women with both PCOS and higher BMI. Chemerin or chemerin receptor plays an essential role in (1) the differentiation pre‐adipocytes to adipocytes by decreasing the expression of adipocyte genes involved in glucose and lipid metabolism^53^, ^54^ and (2) decreasing the aromatase expression which is a key enzyme of sex hormone metabolism and catalyses the conversion of androgens to oestrogens in adipose tissue^55^ and ovarian granulosa cells.^56^ Chemerin decrease both total GLUT4 expression in hGLs and insulin‐induced GLUT4 translocation from the cytoplasm to the membrane. Therefore, chemerin may lead to the attenuation of glucose uptake.^18^ Further, chemerin may play as a negative regulator in FSH‐induced follicular steroidogenesis which may be involved in polycystic ovary morphology (PCOS pathogenesis).^16^, ^56^ The enhancement of FF chemerin may indicate its possible role in stopping follicular growth^10^, ^16^ and ovulatory dysfunction characterizing PCOS pathogenesis.^10^ Granulosa cells have a basic responsibility to provide energy to oocytes for maturation, developmental competency and protection. Granulosa cells (follicular fluid) chemerin can impair through the imbalance of energy substrates (lipid and glucose metabolism) in oocytes.^57^

### 4.1 Limitations and strengths

The limitations of the present systematic review with meta‐analysis include the following: (1) the different in the studies design (the participants with a wide range of BMI (thin to obesity), age, the race/ethnicity, metabolic status (IR, hyperandrogenism and normandrogenism) and disease history) and also the differences in the accuracy and variety of the kits and the methods of assessments used in each study (the various methods of assessment of chemerin levels such as enzyme‐linked immunosorbent assay (ELISA), liquid chromatography/mass spectroscopy‐mass spectroscopy (LC/MS‐MS) and Western blot (Wb) were presented some advantages and disadvantages which can affect the accuracy of the results (Table S9)^49^); (2) heterogeneity among the studies was high, even with subgroup and sensitivity analysis; (3) subgroup analysis was not performed for FF chemerin levels and ovarian chemerin mRNA expression, due to the lack of the number of studies; (4) however, sample size of some studies was small (*n* = 14, 20 and 28),^32^, ^49^ subgroup analysis based on sample size was carried out to address this limitation; (5) despite the use of a comprehensive search strategy, some unexpected findings from some studies may remain unpublished, which may lead to a change in the results; (6) the lack of enough data for chemerin mRNA expression was lead to its evaluation only in the ovary; (7) protein expression of chemerin was not assessed in the present study due to the lack of enough data. Therefore, it was impossible to evaluate whether an enhancement in ovarian chemerin mRNA expression leads to an increase in protein expression (protein expression was evaluated only in two studies (subcutaneous and omental adipose tissues in one study^17^ and granulosa cells in another one)^18^); (8) the quality of few studies were high (*n* = 5; (9), two studies that were found in the search, due to lack of the abstract, full text or every substantial detail, were excluded (Table S3). They might have been included some important data related to this review; and finally, of 22 studies used in the present review, 21 were observational studies (cross‐sectional). Hence, it seems to be necessary to interpret the results for wider applications, cautiously due to the methodological deficiencies of the included studies.

The strengths of the present systematic review include the following (1) the both clinical trials and observational studies were inserted in the analysis; (2) serum chemerin levels were compared in the PCOS and control groups with regard and regardless of BMI or sample size; further, (3) serum chemerin levels were compared in PCOS groups with different BMI. In fact, the comprehensive analyses in the present study were conducted due to further elucidate the potential relationship between chemerin and PCOS. Moreover, there were some strengths in the present meta‐analysis in comparison with the recent meta‐analysis conducted by Lin et al.^20^ In the present study, serum chemerin levels were compared between (a) PCOS and non‐PCOS regardless/regard of BMI (obese/ normal weight) and (b) PCOS women with obesity and normal weight. It is notable that serum chemerin levels were assessed in 19 versus 5 studies which were evaluated in Lin's study.^20^ Moreover, FF chemerin levels and ovarian chemerin mRNA expression were determined between PCOS and non‐PCOS women.

But the weakness of the present study was the assessment of only one adipokine (chemerin) in comparison with Lin's study^20^ which evaluated a panel of adipocytokines (leptin, visfatin, resistin, apelin, omentin, vaspin and irisin).

## 5 CONCLUSION

Serum chemerin levels were remarkably higher in women with PCOS compared to the controls even with subgroup analysis based on BMI or sample sizes. The serum chemerin levels were higher in women with PCOS and higher BMI compared to lower BMI. Moreover, FF chemerin levels and ovarian chemerin mRNA expression were higher in women with PCOS in comparison with women with non‐PCOS. The present findings illustrated that chemerin may be associated with both PCOS status and BMI, independently. It seems to be necessary to design the prospective studies to control the potential confounders such as race, age, ethnicity and history of diseases.

## CONFLICT OF INTEREST

The authors have no conflict of interest.

## AUTHOR CONTRIBUTION

**Anahita Mansoori:** Formal analysis (lead); Software (lead); Writing‐original draft (equal). **Golnaz Amoochi‐Foroushani:** Data curation (supporting); Resources (equal). **Marzie Zilaee:** Conceptualization (supporting); Methodology (equal); Validation (equal); Writing‐original draft (equal); Writing‐review & editing (equal). **Seyed Ahmad Hosseini:** Methodology (equal); Writing‐review & editing (equal). **Maryam Azhdari:** Conceptualization (equal); Data curation (equal); Methodology (supporting); Project administration (supporting); Software (equal); Writing‐original draft (supporting); Writing‐review & editing (supporting).

## ETHICS APPROVAL AND CONSENT TO PARTICIPATE

Not applicable.

## CONSENT FOR PUBLICATION

Not applicable.

## Supporting information

Supplementary Material

## Data Availability

The data that support the findings of this study are available on request from the corresponding author.
