# Supplementary material for: Serum and follicular fluid chemerin and chemerin mRNA expression in women with polycystic ovary syndrome: Systematic review and meta‐analysis
Source: Endocrinol Diabetes Metab. 2021 Oct 26;5(1):e00307. doi: 10.1002/edm2.307 (PMC8754250; doi:10.1002/edm2.307)
Supplement: Supplementary file 1 — Supplementary Material [file EDM2-5-e00307-s001.doc]

**Table S1.** The details of the search strategy for one of the electronic databases (PubMed)

| **PubMed** | **Key words** |
| --- | --- |
| 935 | "Chemerin" |
| 27 | "chemerin protein" |
| 0 | "human TIG2 protein" |
| 0 | "tazarotene induced gene-2 protein" |
| 5 | "retinoic acid receptor responder protein 2" |
| 2 | "RARRES2 protein" |
| 0 | "RAR-responsive protein TIG2" |
| 941 | ("Chemerin"[Title/Abstract] OR "chemerin protein"[Title/Abstract] OR "human TIG2 protein"[Title/Abstract] OR "tazarotene induced gene-2 protein"[Title/Abstract] OR "retinoic acid receptor responder protein 2"[Title/Abstract] OR "RARRES2 protein"[Title/Abstract] OR "RAR-responsive protein TIG2"[Title/Abstract]) |
| 12914 | "Polycystic ovary syndrome" |
| 180 | "polycystic ovary disease" |
| 13127 | "ovary syndrome" |
| 15 | "polycystic syndrome" |
| 13449 | "polycystic ovary" |
| 747 | "stein-leventhal syndrome" |
| 747 | "stein leventhal syndrome" |
| 795 | "stein-leventhal" |
| 2 | "sclerocystic ovarian degeneration" |
| 45 | "ovarian degeneration" |
| 35 | "sclerocystic ovary syndrome" |
| 2881 | "polycystic ovarian syndrome" |
| 2954 | "ovarian syndrome" |
| 37 | "sclerocystic ovaries" |
| 43 | "sclerocystic ovary" |
| 11720 | "PCOS" |
| 17531 | ("Polycystic ovary syndrome"[Title/Abstract] OR "polycystic ovary disease"[Title/Abstract] OR "ovary syndrome"[Title/Abstract] OR "polycystic syndrome"[Title/Abstract] OR "polycystic ovary"[Title/Abstract] OR "stein-leventhal syndrome"[Title/Abstract] OR "stein leventhal syndrome"[Title/Abstract] OR "stein leventhal"[Title/Abstract] OR "sclerocystic ovarian degeneration"[Title/Abstract] OR "ovarian degeneration"[Title/Abstract] OR "sclerocystic ovary syndrome"[Title/Abstract] OR "polycystic ovarian syndrome"[Title/Abstract] OR "ovarian syndrome"[Title/Abstract] OR "sclerocystic ovaries"[Title/Abstract] OR "sclerocystic ovary"[Title/Abstract] OR "PCOS"[Title/Abstract]) |
| 32 | ("Chemerin"[Title/Abstract] OR "chemerin protein"[Title/Abstract] OR "retinoic acid receptor responder protein 2"[Title/Abstract] OR "RARRES2 protein"[Title/Abstract]) AND ("Polycystic ovary syndrome"[Title/Abstract] OR "polycystic ovary disease"[Title/Abstract] OR "ovary syndrome"[Title/Abstract] OR "polycystic syndrome"[Title/Abstract] OR "polycystic ovary"[Title/Abstract] OR "stein-leventhal syndrome"[Title/Abstract] OR "stein-leventhal syndrome"[Title/Abstract] OR "stein-leventhal"[Title/Abstract] OR "sclerocystic ovarian degeneration"[Title/Abstract] OR "ovarian degeneration"[Title/Abstract] OR "sclerocystic ovary syndrome"[Title/Abstract] OR "polycystic ovarian syndrome"[Title/Abstract] OR "ovarian syndrome"[Title/Abstract] OR "sclerocystic ovaries"[Title/Abstract] OR "sclerocystic ovary"[Title/Abstract] OR "PCOS"[Title/Abstract]) |

**Table S2**: Primary identified records through database and other sources searching

| Databases | Number |
| --- | --- |
| Pubmed | 32 |
| Web of ISI | 51 |
| Scopus | 46 |
| Cochrane | 0 |
| Google scholar | 44 |
| Scientific Information Database (SID) | 1 |
| Total | 174 |

**Table S3:** The reasons for the exclusion of the articles after reading full-text.

| **Reference** | **The reason (s) of exclusion** | **Reference** |
| --- | --- | --- |
| (1) | Duplicate data | Tan B et al. (2009) |
| (2) | No full-text: Only abstract file was available.(Lack of the desirable variables in abstract) | Lin et al. (2012) |
| (3) | The studied participant without diagnosed women with PCOS | Luque-Ramı´rez et al. (2013) |
| (4) | Duplicate data | Kort D.H et al. (2013) |
| (5) | No full-text: Only the title of article was available (Google Scholar). | Fu L (2015) |
| (6) | Lack of the desirable variables | Daan N.M.P (2016) |
| (7) | Duplicate data | Rai P (2016) |
| (8) | Lack inclusion criteria | Yang et al. (2018) |
| (9) | Lack of the desirable variables | Morshedzadeh et al. (2019) |

**References:**

1. Tan B, Chen J, Adya R, Kaur J, Heutling D, Lewandowski KC, et al., editors. Insulin increases and metformin decreases the novel adipokine chemerin in insulin resistant subjects: PCOS as a paradigm. Society for Endocrinology BES 2009; 2009: BioScientifica.

2. LIN Y, SUN B, LI X. CHANGES AND SIGNIFICANCE OF SERUM C-REACTIVE PROTEIN, VISFATIN AND chemerin LEVELS IN PATIENTS WITH POLYCYSTIC OVARIAN SYNDROME [J]. Medical Journal of Qilu. 2012;3.

3. Luque-Ramirez M, Martinez-Garcia MA, Montes-Nieto R, Fernandez-Duran E, Insenser M, Alpanes M, et al. Sexual dimorphism in adipose tissue function as evidenced by circulating adipokine concentrations in the fasting state and after an oral glucose challenge. Human Reproduction. 2013;28(7):1908-18.

4. Kort D, Sullivan C, DePinho J, Kostolias A, Ferin M, Lobo R. Chemerin as a marker of body fat and insulin resistance in women with polycystic ovary syndrome (PCOS). Fertility and Sterility. 2013;100(3):S349-S50.

5. Fu L, editor Higher levels of chemerin were detected in the follicular fluids of PCOS than non-PCOS: possibility of involvement of chemerin in the pathogenesis of PCOS. HUMAN REPRODUCTION; 2015: OXFORD UNIV PRESS GREAT CLARENDON ST, OXFORD OX2 6DP, ENGLAND.

6. Daan NM, Koster MP, de Wilde MA, Dalmeijer GW, Evelein AM, Fauser BC, et al. Biomarker Profiles in Women with PCOS and PCOS Offspring; A Pilot Study. PloS one. 2016;11(11):e0165033.

7. RAI P. APPRAISAL OF SERUM CHEMERIN. JOURNAL OF INNOVATION IN BIO SCIENCE. 2016;1(1).

8. Yang X, Quan X, Lan Y, Wei Q, Ye J, Yin X, et al. Serum chemerin level in women with PCOS and its relation with the risk of spontaneous abortion. Gynecological endocrinology : the official journal of the International Society of Gynecological Endocrinology. 2018;34(10):864-7.

9. Morshedzadeh N, Saedisomeolia A, Djalali M, Eshraghian MR, Hantoushzadeh S, Mahmoudi M. Resolvin D1 impacts on insulin resistance in women with polycystic ovary syndrome and healthy women. Diabetes & Metabolic Syndrome-Clinical Research & Reviews. 2019;13(1):660-4.

**Table S4:** Characteristics of the trials included in the meta-analysis

| **Reference** | **PCOS diagnostic criteria*** | **Country** | **PCOS/Control** | **Age **** | **Outcome (unit)** | **Assay approach** | **Quality assessment**# |
| --- | --- | --- | --- | --- | --- | --- | --- |
| **Tan et al. (2009)▀** | Rotterdam and ASRM | England | 14 /14 | 28-38 | Serum chemerin (ng/ml) | ELISA | High |
|  |  |  |  |  | mRNA expression (%) | RT-PCR |  |
|  |  |  |  |  | Protein expression (%) | WB |  |
| **Haghighi et al. (2012)** | NIH | Iran | 45/45 | 18-45 | Serum chemerin (pg/dl) | ELISA | Moderate |
| **Martínez-García et al. (2013)** | Rotterdam | Spain | 7/7 | - | mRNA expression (%) | RT-PCR | Moderate |
| **Martínez-García et al. (2013)** | Rotterdam | Spain | 40/40 | - | Serum chemerin (ng/ml) | ELISA | Moderate |
| **Ademoglu et al. (2014)** | Rotterdam & ASRM | Turkey | 70/38 | 18-33 | Serum chemerin (ng/ml) | ELISA | Moderate |
| **Guzel et al. (2014)** | Rotterdam | Turkey | 80/57 | - | Serum chemerin (ng/ml) | ELISA | Moderate |
| **Kort et al. (2014)** | Rotterdam | Colombia | 45/ 23 | 18-38 | Serum chemerin (ng/ml) | ELISA | Moderate |
| **Wang et al. (2014)** | Rotterdam | China | 67/ 20 |  | Serum chemerin (ng/ml) | ELISA | High |
| **Guducu. et al. (2015)** | Rotterdam & ASRM | Turkey | 35/30 |  | Serum chemerin (ng/ml) | ELISA | High |
| **Huang et al. (2015)** | Rotterdam | China | 148/ 88 |  | Serum chemerin (ng/ml) | ELISA | High |
| **Reda et al. (2015)** | Rotterdam | Egypt | 60/30 | 20-35 | Serum chemerin (ng/ml) | ELISA | Low |
| **Yang et al. (2015)** | Rotterdam | China | 118/114 | 15-35 | Serum chemerin (ng/ml) | ELISA | Moderate |
| **Guvenc etal. (2016)** | Rotterdam | Turkey | 40/30 | 23-34 | Serum chemerin (ng/ml) | ELISA | Moderate |
| **Behboudi‑Gandevani et al. (2017)** | NIH | Iran | 104/58 | 20-40 | Serum chemerin (pg/ml) | ELISA | Moderate |
| **El-Omda et al. (2018)** | Rotterdam | Egypt | 60/20 | 22-37 | Serum chemerin (ng/ml) | ELISA | Moderate |
| **Momenpour et al. (2018)** | Rotterdam | Taipei | 10/10 |  | FF chemerin (μM) | WB | low |
| **Bongrani et al. (2019)** | Rotterdam | France | 51/27 |  | FF chemerin (ng/ml) | ELISA | Moderate |
|  |  |  |  |  | mRNA expression (%) | RT-PCR |  |
| **Foda et al. (2019)** | Rotterdam | Egypt | 100/70 | 21-26 | Serum chemerin (ng/ml) | ELISA | Moderate |
| **Li et al. (2019)** | Rotterdam | China | 51/50 | - | Serum chemerin (ng/ml) | ELISA | Moderate |
|  |  |  |  |  | FF chemerin (ng/ml) | ELISA |  |
|  |  |  |  |  | Protein expression (%) | WB |  |
|  |  |  |  |  | mRNA expression (%) | RT-qPCR |  |
| **Martínez-García et al. (2019)**^▀ ╩^ | Rotterdam | Spain | 17/17 | - | Serum chemerin (ng/ml) | ELISA | Low |
| **Wang et al. (2019)** | Rotterdam | china | 30/23 | 20-35 | FF chemerin (ng/ml) | ELISA | Moderate |
|  |  |  |  |  | mRNA expression (%) | RT-PCR |  |
| **Abruzzese et al. (2020)** | Rotterdam | Argentina | 106/60 | 18-38 | Serum chemerin (ng/ml) | ELISA | High |
| **Halawa et al. (2020)** | Rotterdam | Egypt | 45/45 | 28.93±5.11 | Serum chemerin (ng/dl) | ELISA | Moderate |

ASRM: American Society of Reproductive Medicine; ELISA: Enzyme-Linked Immunosorbent Assay; FF: Follicular fluid; NIH: National Institutes of Health; PCOS: Polycystic ovary syndrome; RT-PCR: Real time- polymerase chain reaction; WB: Western blotting;

* Rotterdam criteria or the revised 2003 Rotterdam European Society for Human Reproduction (ESHRE).

** Mean, median, or range (minimum –maximum) of age total participants is shown in the cell.

**▀** Data at the baseline was used in analysis.

# Quality assessment of the studied was performed by Newcastle–Ottawa (NOS) Quality Assessment Scale for case-control studies and Cochrane Collaboration's tool for clinical trial.

**^╩^** Only this study was a clinical trial, the rest of them were case-control studies.

**Table S5.** Quality assessment of included studies using the Newcastle–Ottawa (NOS) Quality Assessment Scale for case-control studies.

| **First author, year**  **(reference)** | **Selection** | | | | **Exposure** | | | **Comparability** | **Total scores** |
| --- | --- | --- | --- | --- | --- | --- | --- | --- | --- |
|  | adequate case definition | Representativeness of the cases | Community selection of controls | No history of disease among controls | A: secure record for clinical outcome  B: structured interview where blind to case/control status | Same method of ascertainment for cases and controls | Same Non-response rate for cases and controls | A: study controls for age and/or BMI  B: study control for any additional factors |  |
| **Tan et al. (2009)** | * | * | * | - | * | * | - | ** | 7 |
| **Haghighi et al. (2012)** | * | * | - | - | * | * | - | - | 4 |
| **Martínez-García et al. (2013)** | * | * | - | - | * | * | - | ** | 6 |
| **Ademoglu et al. (2014)** | * | * | * | - | * | * | - | * | 6 |
| **Guzel et al. (2014)** | * | * | * | - | * | * | - | - | 5 |
| **Kort et al. (2014)** | * | - | - | - | * | * | - | ** | 5 |
| **Wang et al. (2014)** | * | * | * | * | * | * | - | - | 6 |
| **Guducu. et al. (2015)** | * | * | * | - | * | * | - | ** | 7 |
| **Huang et al. (2015)** | * | * | * | - | * | * | - | ** | 7 |
| **Reda et al. (2015)** | * | * | * | * | * | * | - | * | 7 |
| **Yang et al. (2015)** | * | - | - | - | * | * | - | - | 3 |
| **Guvenc etal. ()2016)** | * | * | * | - | * | * | - | * | 6 |
| **Behboudi‑Gandevani et al. (2017)** | * | * | - | - | * | * | - | ** | 6 |
| **El-Omda et al. (2018)** | * | * | * | - | * | * | - | - | 5 |
| **Momenpour et al. (2018)** | * | - | - | - | * | * | - | - | 3 |
| **Bongrani et al. (2019)** | * | * | * | - | * | * | - | - | 5 |
| **Foda et al. (2019)** | * | * | * | - | * | * | - | - | 5 |
| **Li et al. (2019)** | * | * | * | - | * | * | - | - | 5 |
| **Wang et al. (2019)** | * | * | - | - | * | * | - | ** | 6 |
| **Abruzzese et al. (2020)** | * | * | * | * | * | * | - | ** | 8 |
| **Halawa et al. (2020)** | * | * | - | * | * | * | - | - | 5 |

^#^Low quality ranges: 0-3 stars, moderate quality ranges: 4–6 stars, high quality ranges 7–9 stars.

^◙^This reference is a conference paper without full-text (there is not the complete method in abstract.).

**Table S6:** Cochrane risk of the bias assessment

| **Reference** | **Selection bias** | | **Performance bias** | **Detection bias** | **Attrition bias** | **Reporting bias** | **Overall quality*** |
| --- | --- | --- | --- | --- | --- | --- | --- |
|  | Risk of inadequate generation of a randomized sequence | Risk of inadequate concealment of allocation prior to assignment | Knowledge of allocated intervention by participants and personnel during the study | Knowledge of allocated intervention by outcome assessors | Inadequate assessment of incomplete outcome data | Suggestion of selective outcome reporting |  |
| **Martínez-García et al. (2019)** | High (-1) | High (-1) | High (-1) | Unclear (0) | Low (1) | Low (1) | Low |

* Low quality ranges: −6 to 0, medium quality ranges: 1-3, high quality ranges: 4-6.

**Table S7:** Meta-analysis and leave-one-out sensitivity analysis for the comparison of serum chemerin levels in PCOS with non-PCOS group.

|  | **No. of participants (PCOS /** **non-PCOS group.)** |  | **WMD (95% CI)** |
| --- | --- | --- | --- |
| **Overall effect** | 1191/1065 |  | 12.025 (10.916, 13.133) |
| **Tan et al. (2009)** | 14/14 |  | 32.806(28.267, 37.345) |
| **Haghighi et al. (2012)** | 45/45 |  | 29.694(25.767, 33.620) |
| **Martínez-García et al. (2013)** | 21/20 |  | 30.391 (26.428,34.353) |
| **Martínez-García et al. (2013)** | 19/20 |  | 30.189 (26.228, 34.149) |
| **Ademoglu et al. (2014)** | 36/38 |  | 29.570 (25.636, 33.504) |
| **Guzel et al. (2014)** | 40/27 |  | 32.939 (28.377, 37.502) |
| **Guzel et al. (2014)** | 40/30 |  | 32.702 (28.258, 37.146) |
| **Kort et al. (2014)** | 45/23 |  | 31.378 (27.266, 35.490) |
| **Wang et al. (2014)** | 45/20 |  | 29.617 (25.683, 33.550) |
| **Wang et al. (2014)** | 22/20 |  | 29.974 (26.039, 33.909) |
| **Guducu. et al. (2015)** | 35/30 |  | 30.443 (26.482, 34.405) |
| **Huang et al. (2015)** | 49/88 |  | 31.724 (27.537, 35.912) |
| **Huang et al. (2015)** | 50/88 |  | 31.721 (27.445, 35.999) |
| **Huang et al. (2015)** | 49/88 |  | 30.278 (26.281, 34.275) |
| **Reda et al. (2015)** | 30/12 |  | 28.565 (24.644, 32.487) |
| **Reda et al. (2015)** | 30/18 |  | 28.273 (24.566, 31.979) |
| **Yang et al. (2015)** | 60/55 |  | 30.633 (26.612, 34.654) |
| **Yang et al. (2015)** | 58/59 |  | 30.787 (26.76, 34.815) |
| **Guvenc et al. (2016)** | 40/30 |  | 31.184 (27.172, 35.195) |
| **Behboudi-Gandevani et al. (2017)** | 69/25 |  | 29.871 (25.938, 33.803) |
| **Behboudi-Gandevani et al. (2017)** | 35/33 |  | 30.365 (26.401 34.329) |
| **El-Omda et al. (2018)** | 20/20 |  | 26.730 (22.892, 30.567) |
| **EL-Omda et al. (2018)** | 20/20 |  | 20.240 (16.947,23.534) |
| **Foda et al. (2019)** | 50/35 |  | 28.997 (25.039, 32.954) |
| **Foda et al. (2019)** | 50/35 |  | 25.902 (22.072, 29.732) |
| **Li et al. (2019)** | 25/26 |  | 30.822 (26.817, 34.828) |
| **Li et al. (2019)** | 26/24 |  | 30.206 (26.207, 34.207) |
| **Martínez-García et al. (2019)** | 8/8 |  | 31.328 (27.305, 35.305) |
| **Martínez-García et al. (2019)** | 9/9 |  | 31.308 (27.278, 35.338) |
| **Abruzzese et al. (2020)** | 106/60 |  | 29.130 (25.161, 33.098) |
| **Halawa et al. (2020)** | 27/25 |  | 30.142 (26.140, 34.143) |
| **Halawa et al. (2020)** | 18/20 |  | 30.353 (26.344 , 34.362) |

WMD, Weighted mean differences; 95% CI, 95% confidence intervals; PCOS, polycystic ovary syndrome.

**Table S8.** Egger publication bias test for Serum chemerin levels

| **Outcome** | **No of studies** | **No of Data** | **Coefficient** | **95% CI** | **Standard Error** | **t** | **P** |
| --- | --- | --- | --- | --- | --- | --- | --- |
|  |  |  |  |  |  |  |  |
| **Serum chemerin levels**  (In all studies) | 19 | 32 | 8.72 | 4.9, 12.53 | 1.87 | 4.67 | < 0.001 |
| **Serum chemerin levels**  (In all studies with BMI > 25 or < 25) | 17 | 26 | 5.55 | 1.03, 10.07 | 2.19 | 2.53 | 0.018 |

95% CI, 95% confidence intervals;

**Table S9:** The advantages and disadvantages of some methods of assessment of chemerin.

| Methods | Advantages | Disadvantages |
| --- | --- | --- |
| ELISA | - Determining of the concentration in the ng/mL range | - The lack of diagnosis of different chemerin isoforms - High time-consuming (usually 6 to 8 hours/run) |
| LC/MS-MS | - Quantitative analysis | - Expensive (regular service) |
| Wb | - Specific - Quantitative analysis | - High time-consuming (usually two days) - Expensive |

ELISA: Enzyme-linked immunosorbent assay

LC/MS-MS: Liquid chromatography / mass spectroscopy-mass spectroscopy

Wb: Western blot

**Figure S1.** Forest plot detailing weighted mean differences (WMD) and 95% confidence intervals for the comparison of serum chemerin levels in the PCOS with non-PCOS group.


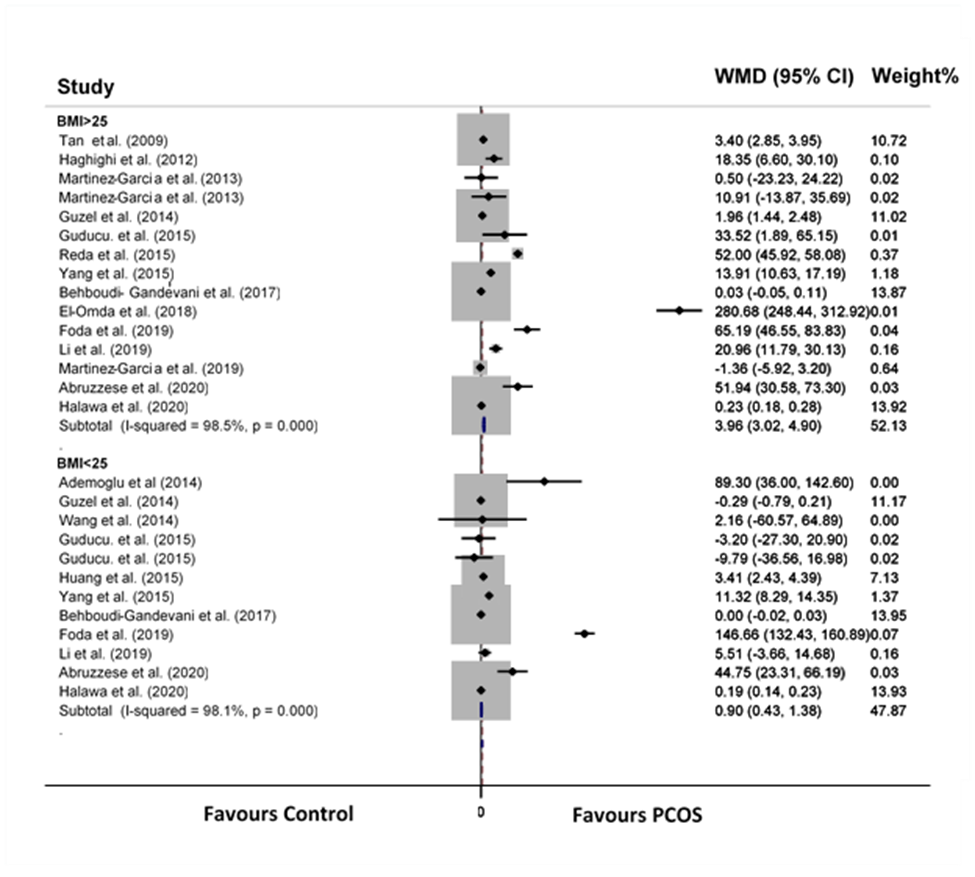


**Figure S2.** Forest plot of subgroup analysis based on BMI>25 or <25 for the comparison of serum chemerin levels in the women with PCOS and non-PCOS.

.

.

Ademoglu et al (2014)

**Sample Size >30**

Martinez-Garcia et al. (2013)

Guzel et al. (2014)

Foda et al. (2019)

Guvenc et al. (2016)

Huang et al. (2015)

Reda et al. (2015)

Foda et al. (2019)

Tan et al. (2009)

Guducu. et al. (2015)

Subtotal (I-squared = 98.4%, p = 0.000)

Halawa et al. (2020)

Yang et al. (2015)

Abruzzese et al. (2020)

Li et al. (2019)

Martinez-Garcia et al. (2019)

El-Omda et al. (2018)

Yang et al. (2015)

Li et al. (2019)

Subtotal (I-squared = 99.5%, p = 0.000)

Wang et al. (2014)

Martinez-Garcia et al. (2019)

EL-Omda et al. (2018)

Halawa et al. (2020)

Reda et al. (2015)

Haghighi et al. (2012)

Behboudi-Gandevani et al. (2017)

Guzel et al. (2014)

Kort et al. (2014)

Martinez-Garcia et al. (2013)

Behboudi-Gandevani et al. (2017)

**Sample Size <30**

89.30 (36.00, 142.60)

10.91 (-13.87, 35.69)

1.96 (1.44, 2.48)

146.66 (132.43, 160.89)

-1.28 (-8.64, 6.08)

3.41 (2.43, 4.39)

52.00 (45.92, 58.08)

65.19 (46.55, 83.83)

3.40 (2.85, 3.95)

-3.20 (-27.30, 20.90)

14.23 (11.03, 17.43)

0.23 (0.18, 0.28)

11.32 (8.29, 14.35)

53.21 (38.71, 67.71)

20.96 (11.79, 30.13)

-1.36 (-5.92, 3.20)

280.68 (248.44, 312.92)

13.91 (10.63, 17.19)

5.51 (-3.66, 14.68)

6.13 (5.25, 7.01)

2.16 (-60.57, 64.89)

0.27 (-3.23, 3.77)

294.25 (279.83, 308.67)

0.19 (0.14, 0.23)

37.70 (35.39, 40.01)

18.35 (6.60, 30.10)

0.03 (-0.05, 0.11)

-0.29 (-0.79, 0.21)

1.43 (0.04, 2.82)

0.50 (-23.23, 24.22)

0.00 (-0.02, 0.03)

0.01

0.05

9.73

0.14

0.51

7.89

0.73

0.08

9.61

0.05

34.04

10.68

2.43

0.14

0.33

1.22

0.03

2.13

0.33

65.96

0.01

1.92

0.14

10.68

3.58

0.20

10.66

9.78

6.22

0.05

10.68

89.30 (36.00, 142.60)

10.91 (-13.87, 35.69)

1.96 (1.44, 2.48)

146.66 (132.43, 160.89)

-1.28 (-8.64, 6.08)

3.41 (2.43, 4.39)

52.00 (45.92, 58.08)

65.19 (46.55, 83.83)

3.40 (2.85, 3.95)

-3.20 (-27.30, 20.90)

14.23 (11.03, 17.43)

0.23 (0.18, 0.28)

11.32 (8.29, 14.35)

53.21 (38.71, 67.71)

20.96 (11.79, 30.13)

-1.36 (-5.92, 3.20)

280.68 (248.44, 312.92)

13.91 (10.63, 17.19)

5.51 (-3.66, 14.68)

6.13 (5.25, 7.01)

2.16 (-60.57, 64.89)

0.27 (-3.23, 3.77)

294.25 (279.83, 308.67)

0.19 (0.14, 0.23)

37.70 (35.39, 40.01)

18.35 (6.60, 30.10)

0.03 (-0.05, 0.11)

-0.29 (-0.79, 0.21)

1.43 (0.04, 2.82)

0.50 (-23.23, 24.22)

0.00 (-0.02, 0.03)

0.01

0.05

9.73

0.14

0.51

7.89

0.73

0.08

9.61

0.05

34.04

10.68

2.43

0.14

0.33

1.22

0.03

2.13

0.33

65.96

0.01

1.92

0.14

10.68

3.58

0.20

10.66

9.78

6.22

0.05

10.68

0

0

Favours Control

Favours PCOS

**Study**

**WMD (95% CI)**

**Weight%**

**Figure S3.** Forest plot of subgroup analysis based on sample size >30 or < 30 for the comparison of serum chemerin levels in the women with PCOS and non-PCOS.

**Figure S4:** Funnel plot for serum chemerin levels for all studies.

**Figure S5:** Funnel plot for serum chemerin levels regarding BMI > 25 or < 25.
